# Uncertainty propagation in financial models of photovoltaic systems

**DOI:** 10.1038/s41598-026-38053-1

**Published:** 2026-02-04

**Authors:** Stefan Wieland, Utku Gürsal

**Affiliations:** Fraunhofer Institute for Solar Energy Systems ISE, Heidenhofstr. 2, 79110 Freiburg, Germany

**Keywords:** Photovoltaics, Economic analysis, Uncertainty propagation, Energy science and technology, Engineering, Mathematics and computing, Physics

## Abstract

Financial analysis has a long history of capturing the stochasticity of real-world phenomena. For informed investment decisions, it is crucial to understand and quantify uncertainty propagation from financial model input to output. Yet to that end, in the photovoltaics sector one has so far relied on coarse-grained approximations or extensive simulations. Here we present a numerically inexpensive approach that exactly traces uncertainty propagation on the level of probability distributions. It leverages analytic shortcuts through switching between different distribution representations, and only assumes independent input variables. With the financial analysis of a typical photovoltaic system as a case study, we use this approach to compute key financial metrics and demonstrate that their values can differ significantly from those obtained by a standard approximation. Moreover, we show with both frameworks that input uncertainty alone can significantly impact the outcome of financial analysis.

## Introduction

A swift and large-scale deployment of renewable energies is essential for limiting global warming^1^, yet it relies on financing. Respective investment decisions however hinge on the computation of profitability metrics such as the net present value (NPV) or levelized cost of electricity (LCOE)^2–4^. These computations in turn need as an input accurate long-term (20+ year) forecasts of often highly fluctuating random variables describing, among others, meteorological conditions, costs and generated yields. The stochastic character of these input variables propagates to calculated economic target variables, and the challenge is to accurately model this uncertainty propagation to enable more informed investment decisions.

As an important pillar for the transition towards renewable energies, photovoltaic (PV) power plants are a popular subject of economic analyses^4–14^. Relevant work on uncertainty propagation in the PV context focuses primarily on LCOE calculation^8,14–17^, but lacks a systematic account of the impact of input uncertainty on the calculation result. One route to achieving the latter is to disentangle the shifting input averages from varying fluctuations around those averages. Our first main contribution presented here is precisely to investigate how input uncertainty alone — while keeping input averages fixed — influences from an investor’s perspective the outcome of LCOE analysis and associated economic metrics. Moreover, we extend this investigation to another key variable, the NPV.

A very coarse-grained way to account for uncertainty propagation in computations are scenario^18,19^ and sensitivity^8,13,14,17,20^ analysis, which are adequate in the absence of both data *on* and educated guesses *for* the random character of input variables. Yet nowadays, the availability of big data more often than not allows for a more detailed characterization of input uncertainty in terms of probability distributions or some of their moments — most notably through averages and standard deviations. In NPV and LCOE computations for PV power plants, random input variables like annual yields or costs are continuous (see Methods) and thus described by probability density functions (PDFs).

In case underlying PDFs are unknown, their averages, standard deviations and percentiles can be well approximated using real-world time series, delivering already useful uncertainty quantifiers. Clearly, NPV and LCOE averages are the primary measures of profitability in NPV and LCOE analysis. Standard deviations, variances and coefficients of variation (CVs) can be interpreted as measures of uncertainty in input and target variables, respectively. In the PV sector, percentiles are used to define thresholds for the respective random variable that are exceeded with a given probability, e.g., the 10th percentile yields *P*90 as a relatively robust lower bound that is exceeded with probability 90%. These deliberate underestimations are a handy metric for investors to assess the bankability of projects. They can also be exactly computed with the random variable’s cumulative distribution function (CDF) *F*(*x*), e.g., by solving $F(P90)=1-90\%$ for *P*90. Other valuable uncertainty measures can only be computed with full information on underlying PDFs, such as the probability $P_\mathrm {\textrm{NPV}> 0}$ that the NPV is positive, i.e., that a given project is profitable.

The guide to the expression of uncertainty in measurement (GUM) lays out the de facto standard of how to define and measure uncertainties, as well as how to trace their propagation from model input to output^21^. It gives approximate equations relating input and output averages as well as respective variances (see equations 3a-3b). These equations, in the following referred to as the *standard approximation* (of uncertainty propagation), generally break down for large standard deviations of nonlinear input variables that can result from a highly intermittent character of renewable energies. Moreover, these equations in their standard form do not capture strongly correlated input variables, but can be amended to account for input correlations. Gaussianity of the target variable is often assumed in literature^9,15,21^ and considered part of the standard approximation here.

For a more detailed treatment of uncertainty propagation, GUM proposes a full mapping of input variable PDFs onto target variable PDFs, yielding all uncertainty measures discussed above as a by-product. For such a mapping, it is straightforward to write down the respective — and generally high-dimensional — integral transforms whose solving GUM advises against, arguing it to be too time-consuming without further simplifications. Instead, the use of Monte-Carlo (MC) simulations is recommended^22^ and indeed pursued in relevant literature for only a handful of input variables^7,8,15,16,23,24^. These stochastic algorithms (i) sample probability distributions of input variables (ii) compute target variables based on sampled input variables and (iii) repeat steps (i)-(ii) to generate probability distributions of target variables. This allows MC simulations to trace uncertainty propagation also for correlated sets of input variables. However, it is difficult to draw analytic conclusions from computed output statistics. Moreover, in order to obtain reliable output statistics, one relies on extensive sampling of input distributions. Achieving acceptable runtimes for MC simulations with dozens of input variables (as in the scenario definitions further below) is beyond the scope of this work and left for future consideration.

Here, as our second main contribution, we extend the aforementioned systematic economic analysis of PV systems to large input uncertainties for which the standard approximation fails. To this end, we present a novel analytic approach that tracks — on the level of entire PDFs — uncertainty propagation in modelling, promising feasible runtimes also for large numbers of input variables. This PDF mapping approach consists of the (mostly numerical) solution of integrals that are of significantly lower dimension than those GUM^22^ puts forward, with the simplification achieved through appropriate conversions between characteristic functions (CFs) as well as PDFs and CDFs. On the one hand, this Accelerating Conversion of Mapping Equations (*ACME*) approach sidesteps the long computation times associated with both MC simulations and brute-force integral transforms while still leaving room for further numerical optimization of involved integrals. On the other hand (and unlike the standard approximation), the presented method is valid for arbitrarily large input uncertainties and delivers the propagation of all PDF moments. The only prerequisite is that of the independence of input variables, which is a common modelling assumption due to scarce data on joint PDFs or even just covariances.

**Fig. 1:**
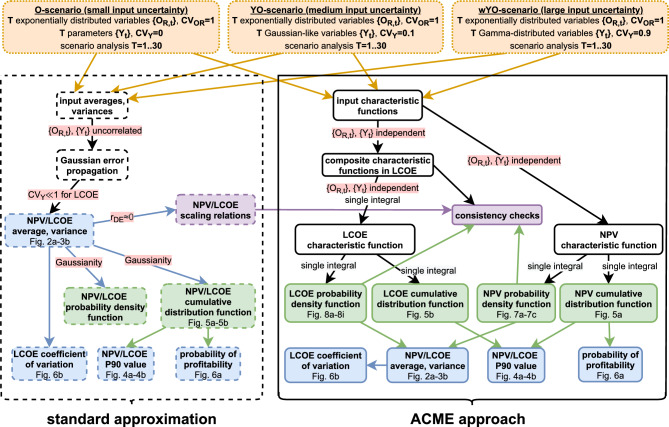
Workflow of and interaction between standard approximation (dashed boxes) and ACME approach (solid boxes) for the three considered scenarios (dotted boxes). Model output – distributions, scalars and equations – is represented and distinguished through shaded boxes. Assumptions used in the workflows are indicated through shaded arrow labels.

In order to systematically analyze uncertainty propagation in the economic forecast for a PV system, we apply the novel approach from an investor’s perspective to multiple scenarios that represent different degrees of input uncertainty, yet constant input averages. To that end, we lay out and motivate in the Methods section the scenarios, as well as relevant input and target variables. These variables are then used to formulate the standard approximation equations and to introduce the proposed ACME formalism. Scaling relations are derived for the dependence of model outputs on a crucial model parameter, and consistency checks for the ACME formalism are formulated to ensure proper numerical implementation. In Results and Discussion, we benchmark both methods using proposed scenarios and a sensitivity analysis, assessing when and how differing degrees of input uncertainty impact key metrics for a PV system’s profitability (cf. Fig. 1).

## Methods

A PV plant’s profitability is influenced by its electric yield, which itself is determined by on-site meteorological conditions such as irradiance, ambient temperatures and wind, but also by technical specifications (e.g., module setup and performance ratio) and the degradation of plant components. Economic factors influencing profitability are the selling price of generated electricity, costs for operation and maintenance (O&M) and investment costs. To assess PV plant profitability in a comparative analysis of ACME approach and standard approximation (cf. Fig. 1), we focus on target variables NPV and LCOE and — without loss of generality — on uncertainty in two types of input variables. This is justified from an investor’s perspective with near-definite knowledge on project-specific input for such an economic analysis, but residual uncertainty tied to environmental variability.

### Target variables

In the PV context, we compute the net present value as

1 $$\begin{aligned} \textrm{NPV}=\sum _{t=1}^{T}{\frac{s\cdot Y_t-O_\textrm{M}-O_{\textrm{R},t}}{\left( 1+r_\textrm{DI}\right) ^t}-I} \end{aligned}$$

which incorporates specific (i.e., normalized by nominal capacity) investment costs *I*, specific time-dependent revenues $s\cdot Y_t$ as well as specific operation and maintenance (O&M) costs $O_\textrm{M}+O_{\textrm{R},t}$ for each year *t* during a PV plant’s lifetime of *T* years. Here $Y_t$ is the specific yield in year *t* sold for a fixed selling price *s*, while O&M costs are split into a constant maintenance-related ($O_\textrm{M}$) and *t*-dependent repair-related ($O_{\textrm{R},t}$) part. The quantity $O_\textrm{M}$ can be assigned a flat rate because it covers routine tasks like monitoring and inspections. Running costs and revenues are discounted with a rate $r_\textrm{DI}$ and, for the sake of simplicity, the residual value of decommissioned plants is not considered here. The levelized cost of electricity

2 $$\begin{aligned} \textrm{LCOE}=\frac{ I+\sum _{t=1}^{T} {\frac{O_M+O_{R,t}}{\left( 1+r_{DI}\right) ^t}}}{ \sum _{t=1}^{T}{ \frac{Y_t}{\left( 1+r_{DI}\right) ^t}}} \end{aligned}$$

gives the average cost of electricity generation over a plant’s lifetime *T*, with the annual specific yields being discounted. With the LCOE, one can rank the competitiveness of different forms of electricity generation independently of electricity monetization, accounting for different plant sizes and cost structures. Unlike the NPV however, it is of limited use when assessing the *absolute* profitability of PV plants. Due to their complementary character in economic analysis, NPV and LCOE are chosen in the following as metrics whose uncertainty propagation from input variables is tracked in three scenarios.

**Table 1:** Input parameters and random variables used in calculations. Upper table section: parameters describing deterministic input. Middle table section: the distribution $P_{\mathrm {O_R}}(x)$ and its parameters describing random input variables $\{O_\textrm{R}\}$. Lower table section: the distribution $P_{\textrm{Y}_t}(x)$ and its parameters describing random input variables $\{Y_t\}$. Boxed quantities are the two parameters varied in the analysis, namely *T* in the sensitivity analysis and $\hat{\sigma }_\textrm{Y}$ in the scenario analysis with small (O), medium (YO) and large (wYO) input uncertainties.

**Quantity**	**Meaning**	**Value**	**Unit**
$\boxed {T}$	PV plant lifetime in years	1..30	1
$r_\textrm{DI}$	Discount rate	0.035	1
$r_\textrm{DE}$	Annual degradation rate	0.005	1
*s*	Electricity selling price	0.2	EUR/kWh
*I*	Specific investment costs	1000	EUR/kWp
$O_\textrm{M}$	Annual specific maintenance-related O&M costs	13	EUR/kWp
$\mu _\mathrm {O_R}$	Mean annual specific repair-related O&M costs	7	EUR/kWp
$\sigma _\mathrm {O_R}$	Standard deviation of annual specific repair-related O&M costs	7	EUR/kWp
$P_\mathrm {O_R}(x)$	PDF of annual specific repair-related O&M costs	$$P_\mathrm {O_R}(x)= {\left\{ \begin{array}{ll} \frac{e^{-x/\mu _\mathrm {O_R}}}{\mu _\mathrm {O_R}} & \text {if } x\ge 0 \\ 0 & \text {otherwise} \end{array}\right. }$$	kWp/EUR
$\hat{\mu }_\textrm{Y}$	Mean annual specific yield before degradation	1000	kWh/kWp
$\boxed {\hat{\sigma }_\textrm{Y}}$	Standard deviation of annual specific yield before degradation	0 (**O**);100 (**YO**); 900 (**wYO**)	kWh/kWp
$\alpha$	Shape parameter of Gamma distribution	$\alpha =\hat{\mu }^2_\textrm{Y}/\hat{\sigma }^2_\textrm{Y}$	1
$\theta _t$	*t*-dependent scale parameter of Gamma distribution	$\theta _t=\hat{\sigma }^2_\textrm{Y}\left( 1-r_\textrm{DE}\cdot t\right) /\hat{\mu }_\textrm{Y}$	kWh/kWp
$P_{\textrm{Y}_t}(x)$	PDF of annual specific yield	$$P_{\textrm{Y}_t}(x)= {\left\{ \begin{array}{ll} \frac{x^{\alpha -1}e^{-x/\theta _t}}{\Gamma (\alpha )\theta _t^\alpha } & \text {if } x\ge 0 \\ 0 & \text {otherwise} \end{array}\right. }$$	kWp/kWh

### Input variables and their use in scenario analysis

The input quantities in NPV and LCOE computation depend on many factors such as PV plant specifications, its geographical location, regional market dynamics, legislation and the pursued business model (in the NPV case). While the validity of the ACME approach does not depend on such specifications, we still assign to input quantities values describing a typical PV plant (see parameters in Table 1), mainly taken from^19^ and largely corroborated by^25^.

In the following, we consider — in three main scenarios for NPV and LCOE computation — uncertainty propagation from two sets of random input variables: *T* annual specific yields $\{Y_t\}$ and *T* annual specific *repair-related* O&M costs $\{O_{\textrm{R},t}\}$, with $t=1..T$. In these scenarios, any uncertainty in computed NPVs and LCOEs arises through uncertainty in these input variables, which are considered on an annual basis *t*, as this is the temporal resolution on which standard NPV and LCOE operate [see equations (1)-(2)]. However, this input uncertainty does not refer to the random character of inter-annual variability as in other works^26^. Instead, each year *t* of operation in equations (1)-(2) is given its own pair $\{Y_t,O_{\textrm{R},t}\}$ of random input variables, reflecting respective uncertainties in year *t*, but not beyond. As in other works, it is assumed here that all random input variables are independent, which implies zero correlations within any pair of input variables.

The choice of these two sets of random input variables is motivated by the following observations: (i) In the planning stage for PV plants, investors and project developers usually have specific information on quantities like *I*, $O_\textrm{M}$, $r_\textrm{DE}$ and $r_\textrm{DI}$ which are typically fixed over the project lifetime. Yet they need to accept and capture the stochasticity of yield generation and failure occurence [cf. $P_{\textrm{Y}_t}(x)$ and $P_\mathrm {O_R}(x)$ in Table 1]. (ii) The random character of these latter two processes is quantified in literature. (iii) They have markedly different PDFs, allowing for non-trivial behavior of the calculated target PDFs (see below). Correlations are neglected in all calculations of this work, an assumption that could however be relaxed (see Methods section).

To systematically analyze uncertainty propagation in NPV and LCOE calculation, we choose a sequence of scenarios that represents different degrees of uncertainty in the set $\{Y_t\}$ of *T* annual yields, while keeping uncertainty in $\{O_{\textrm{R},t}\}$ as well as all averages and other parameters constant across scenarios. This is to investigate the impact of input uncertainty on the outcome of economic analysis, and to disentangle in the process the contribution of either set {$O_{\textrm{R},t}$} and {$Y_t$}. In the **O-scenario**, we assume deterministic $Y_t$ and thus set $\hat{\sigma }_Y=0$, but draw *T* random variables {$O_{\textrm{R},t}$} ($t=1..T$) from a *t*-independent exponential distribution $P_\mathrm {O_R}(x)$ (cf. Table 1). Consequently, both NPV and LCOE are linear combinations of *T* independent and identically distributed random variables as also frequently assumed in standard literature. This is used as a base scenario to verify derived expressions, as well as to assess the propagation of the non-Gaussianity in $\{O_{\textrm{R},t}\}$ to the respective target variable. In the **YO-scenario**, the *T* exponentially distributed $\{O_{\textrm{R},t}\}$ ($t=1..T$) are combined with another set $\{Y_t\}$ ($t=1..T$) of *T* random variables drawn from a *t*-dependent narrow Gamma distribution $P_{\textrm{Y}_t}(x)$ (cf. Table 1). This incorporation of non-identically distributed variables in linear (NPV) or nonlinear (LCOE) expressions reflects our current understanding of input uncertainties in LCOE and NPV computation. In the **wYO-scenario**, the same 2*T* independent random input variables as in the YO-scenario are considered, but the Gamma distribution underlying $\{Y_t\}$ is widened considerably. This describes a strongly fluctuating annual yield (around the same average as in the YO-scenario) induced through pronounced climate volatility.

Used input PDFs and their parameters in Table 1 can be motivated both on theoretical and empirical grounds. On the theoretical side, the employed Gamma distribution with shape parameter $\alpha$ and *t*-dependent scale parameter $\theta _t$ is highly versatile, with the exponential and normal distribution as limiting cases for $\alpha =1$ and $\alpha \rightarrow \infty$, respectively. These limiting cases are indeed observed for $\{O_{\textrm{R},t}\}$ and $\{Y_t\}$, see below. Moreover, the Gamma distribution has support $[0,\infty )$, accounting for the fact that both $O_{\textrm{R},t}$ and $Y_t$ are non-negative for all considered *t*. Lastly, it has a very simple characteristic function $\varphi (f)=(1-i\cdot f \cdot \theta _t)^{-\alpha }$ that allows for quick algebraic manipulation and numeric integration in the ACME approach.

Experimentally, the choice of the PDF for $O_{\textrm{R},t}$ is motivated by a recent comprehensive study involving around 80 PV rooftop systems^27^. There, a *t*-independent exponential distribution with mean $\mu _\mathrm {O_R}$ and standard deviation $\sigma _\mathrm {O_R}$ of $7 \, \mathrm {EUR/kW_p}$ is found to sufficiently capture data variability, which is reproduced here by setting the Gamma distribution’s shape parameter to $\alpha =1$ and its scale parameter to $\theta =7 \, \mathrm {EUR/kW_p}$. Other studies quantify uncertainty in overall annual O&M costs ($O_{\textrm{R}_t}+O_\textrm{M}$), assigning a normal distribution^8,16^ or uniform distribution $[17]$, with CVs ranging from 0.05^8^ to 0.33^16^. Yet in those cases, the chosen probability distributions seem to stem from the maximum-entropy principle rather than from sampling real-world cost PDFs.

For $Y_t$, a normal distribution is commonly assumed^16,28,29^ or deemed plausible^8^; but see^17^ that operates with an exponential shape instead. The YO-scenario approximates this normal distribution, as there the Gamma distribution’s shape parameter $\alpha =100$ is fairly large, with the associated CV of 0.1 in line with^16^. The wYO-scenario with its shape parameter of roughly $\alpha =1$ and CV of 0.9 resembles the setup with an exponentially distributed capacity factor in^17^, while the O-scenario approximates the very narrow normal distribution with CV=0.03 used in^8^ (cf. Table 1 and Fig. 1).

Note that, to account for module degradation, the Gamma-distributed $Y_t$ have a time-dependent mean $\mu _\textrm{Y}(t)=\hat{\mu }_\textrm{Y}\left( 1-r_\textrm{DE}\cdot t\right)$ and standard deviation $\sigma _\textrm{Y}(t)=\hat{\sigma }_\textrm{Y}\left( 1-r_\textrm{DE}\cdot t\right)$ with the scenario-specific initial annual specific yield $\hat{\mu }_\textrm{Y}$ and standard deviation $\hat{\sigma }_\textrm{Y}$, as well as the annual linear degradation rate $r_\textrm{DE}$^30^ (see Table 1). Both the mean and the standard deviation are set to decrease by the same fraction in each year *t*. This is because small averages usually entail small variability and, without further data, a constant coefficient of variation in $Y_t$ is a sensible guess. Consequently, $\alpha$ is a constant, while $\theta _t$ depends on *t* (cf. Table 1).

### Standard approximation

Formalizing notation in uncertainty propagation, one deals with a vector $\textbf{x}=(x_1, x_2,..., x_v)$ of input variables — with associated mean values $\mathbf {\mu _x}=\left( \mu _1, \mu _2,..., \mu _v\right)$ and standard deviations $\mathbf {\sigma _x}=\left( \sigma _1, \sigma _2,...,\sigma _v\right)$ — feeding into the computation of a target variable $f(x_1, x_2,...,x_v)$ (e.g., the LCOE or NPV). If $f=\sum _{j=1}^v{a_j x_j}$ is a mere linear combination of *uncorrelated* input variables, then its variance — the squared standard deviation — is $\sigma _f^2=\sum _{j=1}^v{a_j^2 \sigma _j^2}$. This is similar to the propagation of averages $\mu _f=\sum _{j=1}^v{a_j \mu _j}$ for such a linear function *f*. If, instead, *f* factorizes as $f=k\prod _{j=1}^v{x_j}$ with *independent* input variables and constant *k*, then $\sigma _f^2=k^2\left[ \prod _{j=1}^v{\left( \sigma _j^2+\mu _j^2\right) -\prod _{j=1}^v\mu _j^2}\right]$ since *f*’s raw moments also factorize. The latter re-declaration of a given output variable *f* as a product of input variables is a common procedure in literature to simplify the computation of $\sigma _f$^15,24^. However, it shifts the modeller’s efforts towards interpreting the associated input variables $x_j$ and quantifying their standard deviations $\sigma _j$.

For general functional forms $f(x_1, x_2,...,x_v)$, the standard approximation is to consider their Taylor expansion around $\textbf{x}=\mathbf {\mu _x}$, assuming uncorrelated input variables $x_j$^21^. This delivers output averages and variances as

3a $$\begin{aligned} \mu _f&\approx f\left( \mu _1,\mu _2,...,\mu _v\right) +\frac{1}{2}\sum _{j=1}^v{\frac{\partial ^2 f}{\partial x_j^2} \biggr |_\mathrm {\textbf{x}=\mathbf {\mu _x}} \sigma _j^2}\end{aligned}$$

3b $$\begin{aligned} \sigma _f^2&\approx \sum _{j=1}^v{\left( \frac{\partial f}{\partial x_j}\biggr |_\mathrm {\textbf{x}=\mathbf {\mu _x}}\right) ^2 \sigma _j^2} \, , \end{aligned}$$

where only terms up to $O(\sigma _j^2)$ are considered. Note that already the approximate equation for the average yields a counter-intuitive second-order term which can however be motivated through a simple example: consider the function $f(x)=x^2$ of a single random variable *x* with mean $\mu _x\equiv \langle x \rangle$ and variance $\sigma ^2_x\equiv \langle x^2 \rangle -\langle x \rangle ^2$, where $\langle \cdot \rangle$ denotes averaging. From $\mu _f\equiv \langle f \rangle =\langle x^2 \rangle$ follows $\mu _f=f(\mu _x)+\sigma _x^2$, i.e., $\mu _f= f\left( \mu _x\right) +\frac{1}{2}\frac{\partial ^2 f}{\partial x^2} \biggr |_{{\textbf {x}}={\mu _{{\textbf {x}}}}} \sigma _x^2$ as an exact equality. The equation for the variance is known as the Gaussian law of error propagation and, for the special case of $f=k\prod _{j=1}^v x_j$, turns into $\sigma ^2_f/f^2\approx \sum _{j=1}^v\sigma ^2_j/x_j^2$, directly relating coefficients of variation instead of absolute standard deviations, which is the de-facto standard expression in the PV sector for analytically calculating uncertainty propagation^9,15^.

Reassuringly, these equations are completely agnostic with respect to the shape of underlying PDFs, including the symmetry of the latter. And yet, assuming Gaussian input variables further simplifies calculations: The target variable *f* is Gaussian if it is a linear combination of independent Gaussian input variables. This is useful, because in the Gaussian case, standard deviations can be quickly converted to percentiles through standard normal tables. These simplifications add to the appeal of using Gaussian variables, but also let some modelers mistake their usefulness for their necessity.

For averages and variances of NPVs and LCOEs, equations 3a-3b yield

4a $$\begin{aligned} \mu _\textrm{NPV}&=\sum _{t=1}^{T}{\frac{s \cdot \hat{\mu }_\textrm{Y}\left( 1-r_\textrm{DE}\cdot t\right) -\mu _\mathrm {O_R}-O_\textrm{M}}{\left( 1+r_\textrm{DI}\right) ^t}-I}\end{aligned}$$

4b $$\begin{aligned} \sigma ^2_\textrm{NPV}&=\sum _{t=1}^T{\frac{s^2\hat{\sigma }^2_\textrm{Y}\left( 1-r_\textrm{DE}\cdot t\right) ^2+\sigma ^2_\mathrm {O_R}}{\left( 1+r_\textrm{DI}\right) ^{2t}}} \end{aligned}$$

4c $$\begin{aligned} \mu _\mathrm {\textrm{LCOE}}&\approx \frac{ I+\sum _{t=1}^{T} { \frac{O_\textrm{M}+\mu _\mathrm {O_R}}{\left( 1+r_\textrm{DI}\right) ^t} } }{ \sum _{t=1}^{T} { \frac{\hat{\mu }_\textrm{Y}\left( 1-r_\textrm{DE}\cdot t\right) }{\left( 1+r_\textrm{DI}\right) ^t} } } \Biggl \{ 1+ \frac{ \sum _{t=1}^{T} { \frac{\hat{\sigma }^2_\textrm{Y}\left( 1-r_\textrm{DE}\cdot t\right) ^2}{\left( 1+r_\textrm{DI}\right) ^{2t}} } }{ \left[ \sum _{t=1}^{T} { \frac{\hat{\mu }_\textrm{Y}\left( 1-r_\textrm{DE}\cdot t\right) }{\left( 1+r_\textrm{DI}\right) ^t} }\right] ^2 }\Biggl \}\end{aligned}$$

4d $$\begin{aligned} \sigma ^2_\textrm{LCOE}&\approx \frac{ \sum _{t=1}^{T} {\left( 1+r_\textrm{DI}\right) ^{-2t}} }{ \left[ \sum _{t=1}^{T} { \frac{\hat{\mu }_\textrm{Y}\left( 1-r_\textrm{DE}\cdot t\right) }{\left( 1+r_\textrm{DI}\right) ^t} }\right] ^2 } \sigma _\mathrm {O_R}^2 + \frac{\left[ I+\sum _{t=1}^{T} { \frac{O_\textrm{M}+\mu _\mathrm {O_R}}{\left( 1+r_\textrm{DI}\right) ^t} }\right] ^2 }{ \left[ \sum _{t=1}^{T} { \frac{\hat{\mu }_\textrm{Y}\left( 1-r_\textrm{DE}\cdot t\right) }{\left( 1+r_\textrm{DI}\right) ^t} }\right] ^4 } \sum _{t=1}^T { \frac{\hat{\sigma }^2_\textrm{Y}\left( 1-r_\textrm{DE}\cdot t\right) ^2}{\left( 1+r_\textrm{DI}\right) ^{2t}} } \,, \end{aligned}$$

where only equations (4a)-(4b) are exact due to the linearity of the NPV in its considered input variables [cf. equation (1)]. Additionally assuming a Gaussian target distribution parametrized by the calculated averages and variances, the standard approximation equations (4a)-(4d) can be used to estimate $P_\mathrm {\textrm{NPV}> 0}$ as well as P90 values. Note that equations (4a)-(4d) are also valid in the O-scenario (setting $\sigma _\textrm{Y}=0$) and, in that case, moreover all exact [due to the linear dependence of equations (1)-(2) on $O_{\textrm{R},t}$].

### ACME approach

The proposed PDF mapping is a two-step process, where the second step is necessary only for nonlinear target variable LCOE:

1. In the functional form of the target variable, any linear combination $\sum _i a_i X_i$ of independent input variables $X_i$ (with possibly different PDFs) is expressed as a new composite variable *Z*. The characteristic function of *Z*, which is defined as the Fourier transform of *Z*’s PDF, is then simply $\varphi _Z (f)=\prod _i{\varphi _i\left( a_i f\right) }$, where $\varphi _i(f)$ is the CF of input variable $X_i$. Introducing such composite variables significantly reduces complexity through replacing multiple integration of PDFs in probability space with mere multiplication of CFs in Fourier space. This already delivers the NPV PDF and CDF through a single numerical integration (with the respective Gil-Pelaez inversion formula) in all considered scenarios and the LCOE PDF in the O-scenario, since — according to equations (1)-(2) — we deal in those cases with just a linear combination of (assumed independent) input variables. In contrast, the brute-force strategy laid out in GUM^22^ requires — both for obtaining NPV and LCOE PDFs — solving high-dimensional integrals over the PDFs of $T=30$ (O-scenario) or $2T=60$ (YO- and wYO-scenario) random variables.
2. For the target variable LCOE in the YO- and wYO-scenario that is a nonlinear function of random variables, PDF and CDF cannot be exclusively computed with step 1. Instead, both numerator and denominator are expressed as composite random variables $\hat{Z}$ and $Z_1$, respectively, according to step 1. Both are measurable functions of disjoint sets of independent random variables and thus also independent. This allows to write the CF of LCOE as $\varphi _\textrm{LCOE}(f)\equiv \varphi _{\hat{Z}/Z_1}(f)=\int _{-\infty }^\infty {\textrm{d}z_1\,\varphi _\mathrm {\hat{Z}}\left( f/z_1\right) P_\mathrm {Z_1}(z_1)}$, where $\varphi _\mathrm{\hat{Z}}(f)$, $\varphi _\mathrm {Z_1}(f)$ and $P_\mathrm {Z_1}(z_1)$ are obtained as in step 1. Finally, the PDF and CDF of the LCOE are computed from $\varphi _\textrm{LCOE}(f)$. Hence in total, two single integrations are necessary in this case to compute LCOE distributions.

#### Computation of NPV distributions in ACME approach

We first detail the NPV PDF computation for the YO- and wYO-scenario, and then adapt obtained expressions to the O-scenario. According to equation (1), one can write $\textrm{NPV}=s \cdot Z_1-Z_2-Z_3$ with composite variables $Z_1\equiv \sum _{t=1}^T{Y_t/(1+r_\textrm{DI})^t}$ and $Z_2\equiv \sum _{t=1}^T{O_{\textrm{R},t}/(1+r_\textrm{DI})^t}$ as well as constant $Z_3\equiv I+O_\textrm{M}\sum _{t=1}^T{(1+r_\textrm{DI})^{-t}}$. The CF of the annual yield $Y_t$ is $\varphi _{\textrm{Y}_t}(f)=\left[ 1- i\cdot f\cdot \hat{\sigma }^2_\textrm{Y}/\hat{\mu }_\textrm{Y} \left( 1-r_\textrm{DE}\cdot t\right) \right] ^{-\hat{\mu }^2_\textrm{Y}/\hat{\sigma }^2_\textrm{Y}}$, the CF of $O_{\textrm{R},t}$ is $\varphi _{\textrm{O}_\textrm{R,t}}(f)=\left( 1-i\cdot f \cdot \sigma _\mathrm {O_R} \right) ^{-1}$, and the CF of any constant *c* is $\varphi _\textrm{c}(f)=e^{i\cdot f\cdot c}$. Therefore the CFs of $Z_1$, $Z_2$ and $Z_3$ are, according to step 1 of the PDF mapping approach,

5a $$\begin{aligned} \varphi _\mathrm {Z_1}(f)&=\left( \prod _{t=1}^T{\left[ 1- i\cdot f\frac{\hat{\sigma }^2_\textrm{Y} }{\hat{\mu }_\textrm{Y}} \frac{\left( 1-r_\textrm{DE}\cdot t\right) }{\left( 1+r_\textrm{DI}\right) ^t} \right] }\right) ^{-\hat{\mu }^2_\textrm{Y}/\hat{\sigma }^2_\textrm{Y}} \end{aligned}$$

5b $$\begin{aligned} \varphi _\mathrm {Z_2}(f)&=1/\prod _{t=1}^T{\left[ 1-i\cdot f\frac{\sigma _\mathrm {O_R}}{(1+r_\textrm{DI})^t}\right] } \end{aligned}$$

5c $$\begin{aligned} \varphi _\mathrm {Z_3}(f)&=\exp {\left[ i\cdot f \cdot Z_3\right] } \end{aligned}$$

The CF of the NPV is simply the product

6 $$\begin{aligned} \varphi _\textrm{NPV}(f)=\varphi _\mathrm {Z_1}(s\cdot f)\cdot \varphi _\mathrm {Z_2}(-f)\cdot \varphi _\mathrm {Z_3}(-f) \, , \end{aligned}$$

again according to equation (1) and the fact that also $Z_1$ and $Z_2$ are independent, being measurable functions of disjoint sets of independent random variables. The Gil-Pelaez inversion formulas then yield for the NPV PDF

7 $$\begin{aligned} P_\textrm{NPV}(x)=\frac{1}{\pi }\int _\textrm{0}^\infty {\textrm{d}f\,\operatorname {Re}\left[ e^{-i\cdot f\cdot x}\varphi _\textrm{NPV}(f)\right] } \end{aligned}$$

and $F_\textrm{NPV}(x)=1/2-\pi ^{-1}\int _\textrm{0}^\infty {\textrm{d}f\,f^{-1}\operatorname {Im}\left[ e^{-i\cdot f\cdot x}\varphi _\textrm{NPV}(f)\right] }$ for the NPV CDF. Here $\operatorname {Re}[z]$ and $\operatorname {Im}[z]$ are real and imaginary part of complex number *z*, respectively.

For the O-scenario, we set instead

8 $$\begin{aligned} \varphi _\textrm{NPV}(f)=\varphi _\mathrm {Z_2}(-f)\cdot \exp {\left[ i\cdot f \left( s\cdot \langle Z_1\rangle -Z_3\right) \right] } \end{aligned}$$

and compute PDF and CDF as above. Here $\langle . \rangle$ is the ensemble average, so that $\langle Y_{t}\rangle =\hat{\mu }_\textrm{y}(1-r_\textrm{DE}\cdot t)$ in $\langle Z_1\rangle$.

#### Computation of LCOE distributions in ACME approach

For the YO- and wYO-scenario, we set $\hat{Z}\equiv Z_2+Z_3$ and observe $\textrm{LCOE}=\hat{Z}/Z_1$ [cf. equation (2]. We then calculate the characteristic functions of $\hat{Z}$ [delivering $\varphi _\mathrm {\hat{Z}}(f)=\varphi _\mathrm {Z_2}(f)\cdot \varphi _\mathrm {Z_3}(f)$] and $Z_1$ [yielding $\varphi _\mathrm {Z_1}(f)$]. Knowing that $P_{\textrm{Y}_t}(0)=0$ in all scenarios due to $\alpha>1$ (cf. Table 1), it follows that also $P_\mathrm {Z_1}(0)=0$, so that

9 $$\begin{aligned} \varphi _\textrm{LCOE}(f)=\int _{0^+}^\infty {\textrm{d}z_1\,\varphi _\mathrm {Z_2}\left( f/z_1\right) \cdot \varphi _\mathrm {Z_3}\left( f/z_1\right) \cdot P_\mathrm {Z_1}(z_1)}\,. \end{aligned}$$

For the LCOE PDF computation in the O-scenario, we proceed similarly to the respective NPV calculation, obtaining

10 $$\begin{aligned} \varphi _\textrm{LCOE}(f)=\varphi _\mathrm {Z_2}(f/\langle Z_1\rangle )\cdot \exp {\left[ i\cdot f \cdot Z_3 / \langle Z_1\rangle \right] }\,. \end{aligned}$$

The LCOE PDF and CDF are then obtained from the CF analogously to the NPV case.

### Understanding the temporal scaling

In our systematic analysis of uncertainty propagation further below, all computed quantities are subject to an additional sensitivity analysis with respect to the plant lifetime *T*. This is because uncertainty propagation from *T* to target variables NPV and LCOE, with *T* being a discrete model parameter, cannot be traced with ACME or standard approach in their form laid out above. Still, uncertainty in *T* can be considerable due to environmental or economic hazards, and thus should be accounted for. Here, we qualitatively predict — through approximate scaling relations — benchmarking results in Results and Discussion for the *T*-dependent behavior of computed quantities. To this end, we make use of the fact that both the discount rate $r_\textrm{DI}$ and degradation rate $r_\textrm{DE}$ commonly attain very small values (see Table 1).

To assess the *T*-dependence of computed averages and standard deviations, we use equations (4a)-(4d). For small $r_\textrm{DI}$ and $r_\textrm{DE}$, we obtain

11a $$\begin{aligned} \mu _\mathrm {\textrm{NPV}}(T)&\approx T \left( s \cdot \hat{\mu }_\textrm{Y}-\mu _\mathrm {O_R}-O_\textrm{M}\right) -I\end{aligned}$$

11b $$\begin{aligned} \sigma ^2_\mathrm {\textrm{NPV}}(T)&\approx T \left( s^2\hat{\sigma }_\textrm{Y}^2+\sigma ^2_\mathrm {O_R}\right) \end{aligned}$$

11c $$\begin{aligned} \mu _\mathrm {\textrm{LCOE}}(T)&\approx \frac{ I/T+O_\textrm{M}+\mu _\mathrm {O_R} }{ \hat{\mu }_\textrm{Y} } \Biggl \{ 1+ \frac{ \hat{\sigma }^2_\textrm{Y} }{ T\cdot \hat{\mu }_\textrm{Y}^2 }\Biggl \}\end{aligned}$$

11d $$\begin{aligned} \sigma ^2_\mathrm {\textrm{LCOE}}(T)&\approx \frac{ \sigma _\mathrm {O_R}^2 }{ T\cdot \hat{\mu }_\textrm{Y}^2 } + \frac{ \left[ T^{-3/2}I+T^{-1/2}\left( O_\textrm{M}+\mu _\mathrm {O_R}\right) \right] ^2 }{ \hat{\mu }_\textrm{Y}^4 }\hat{\sigma }_\textrm{Y}^2 \,. \end{aligned}$$

In these simplified standard approximation equations, the averages’ and variances’ dependence on *T* can be read off easily and compared to predictions of the ACME approach and the original equations (4a)-(4d) (see Results and Discussion).

We further note that the LCOE equations (4c)-(4d) are exact only in the O-scenario and approximate in the YO- and wYO-scenario. To understand how *T* influences the quality of latter approximations, we first remark that for $r_\textrm{DE}\rightarrow 0$, $P_{\textrm{Y}_t}(x)$ is *t*-independent (cf. Table 1). With now all input distributions being *t*-independent as well as $r_\textrm{DI}\rightarrow 0$, we rewrite equation (2) as

12 $$\begin{aligned} \textrm{LCOE}(T)\approx \frac{ \langle O_{\textrm{R},t}\rangle _T + O_\textrm{M}+ I/T }{ \langle Y_t \rangle _T } \, . \end{aligned}$$

Here $\langle Y_t \rangle _T=T^{-1}\sum _{t=1}^T Y_t$ and $\langle O_{\textrm{R},t}\rangle _T=T^{-1}\sum _{t=1}^T O_{\textrm{R},t}$ are sample means (with sample size *T*) of random variables $Y_t$ and $O_{\textrm{R},t}$, respectively. These sample means are themselves random variables drawn from *T*-dependent distributions with variances $\hat{\sigma }_\textrm{Y}^2/T$ and $\sigma _\mathrm {O_R}^2/T$, respectively. Consequently, the LCOE output variable can be approximated as a function of only two random input variables $\langle Y_t \rangle _T$ and $\langle O_{\textrm{R},t}\rangle _T$ whose variances decrease with *T* (with the only other LCOE dependence on *T* given by a vanishing additive term in the numerator of the LCOE). This suggests that the accuracy of the standard approximation equations for LCOE averages and standard deviations will increase with *T*.

### Consistency checks in ACME approach

Given independent input variables, the presented ACME approach is exact, but needs careful numerical implementation. This is ensured by the following consistency checks on ACME output:

Computed PDFs of NPV and LCOE (as well as of all input and intermediate characteristic functions) must be non-negative on considered intervals. The area under each of these PDF curves must moreover be 1 on considered intervals. NPV averages and variances computed from equation (7) must match those in equations (4a)-(4b). Additionally, LCOE averages and variances computed from the inversion formula applied to equation (10) in the O-scenario should match those in equations (4c). Furthermore, averages and variances computed from $P_\mathrm {\hat{Z}}(x)$ and $P_\mathrm {Z_1}(x)$ must match those obtained from the numerator and denominator in equation (2), respectively (as both $\hat{Z}$ and $Z_1$ are also linear in considered input variables). ACME averages and variances should qualitatively obey scaling relations given by equations (11a)-(11d). LCOE averages and variances computed in the ACME approach should coincide with their standard approximation counterparts (i) for small input variances (i.e., in the O- and YO-scenario) as well as (ii) for large *T* (in any scenario). Moreover, for large *T*, the NPV PDFs must be Gaussian to a high degree of accuracy, since then in equation (1), the central limit theorem approximately holds due to $r_\textrm{DI}\approx 0$ and $r_\textrm{DE}\approx 0$. This also applies to the LCOE PDF in the O-scenario [cf. equation (2)].

### Correlated input variables

Some frameworks competing with ACME can in principle incorporate input covariances, but in practice assume uncorrelated input variables^9,15,21–23^. Like^24^, the ACME framework relies on the stronger assumption of independent input variables which, given the lack of data on respective joint probabilities, is a sensible approach from an operational perspective. But ACME’s independence assumption translates into strict real-world requirements for the modeled PV system: First, assuming independent annual yields $\{Y_t\}$ implies neglecting inter-annual climate trends. Second, independent repair-related operation and maintenance costs $\{O_{\textrm{R},t}\}$ presuppose that PV system failure events appear independently of failures and repairs in previous years. Third, imposing independent $\{Y_{t_1},O_{\textrm{R},t_2}\}$ (with $t_1,t_2=1..T$) assumes that losses in $Y_t$ (due to failure- and repair-induced PV system downtime) are balanced out by repair-induced gains in $Y_t$ due to higher system performance. These requirements are hardly realistic, so it is natural to ask how the ACME approach can be adapted to account for input correlations in case these are known. One obvious strategy targets the summation over *t* in equations (1)-(2): Instead of binning all summed input variables into a single composite random variable as in the original approach, only those with sufficiently large time lags are pooled together to minimize cross-correlations between them — for example, only those with an even index *t* into one composite variable and those with an odd index *t* into another. This results in NPV and LCOE expressions with potentially very few significantly correlated composite variables, with higher chances of proper analytical or numerical treatment than the initial expressions.

## Results and discussion

With both uncertainty propagation frameworks laid out, input and target variables specified as well as scenarios defined, we want to use these to systematically trace uncertainty propagation in the NPV and LCOE analysis of PV plants (cf. Fig. 1). To that end, equations (4a)-(4d) from the standard approximation are used to compute (mostly approximate) quantities that are then contrasted with their exact counterparts obtained from the ACME approach. These are NPV and LCOE averages, standard deviations as well as *P*90 values. To explore the behaviour of P90 values, we additionally plot cumulative distribution functions. Moreover, we consider $P_\mathrm {\textrm{NPV}\ge 0}$ (the probability of profitability) as well as the LCOE coefficient of variation. The respective ACME output in the YO- and wYO-scenario is obtained from equations (6) and (7) (NPV) as well as the Gil-Pelaez inversion of equation (9) (LCOE). In the O-scenario, equations (7) and (8) (NPV) as well as the Gil-Pelaez inversion of equation (10) (LCOE) are used, with all ACME output subject to the consistency checks laid out above. Plotting the underlying ACME PDFs, we assess their degree of Gaussianity to evaluate whether and when Gaussianity is a justified assumption in simplified models like the standard approximation.

### Averages

**Fig. 2:**
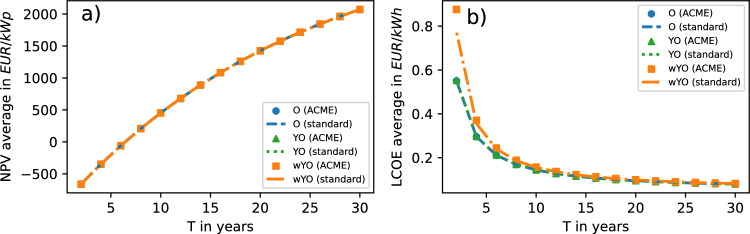
(**a**) NPV averages and (**b**) LCOE averages computed with ACME approach and standard approximation in three scenarios.

As shown in Fig. 2a, NPV averages increase roughly linearly with the number of years of operation *T* [cf. equation (11a)]. Moreover, since the NPV is linear in considered input variables, the latter variables’ variances do not affect computed NPV averages — hence NPV curves for the O-, YO- and wYO-scenario coincide. This linearity furthermore renders equation (4a) exact, letting NPV curves for the standard approximation and for the ACME approach coincide as well.

As expected [cf. equation (11c)], LCOE averages decrease with *T* (Fig. 2b). For large *T*, LCOE averages converge across scenarios, as then the only scenario-specific term $\hat{\sigma }^2_\textrm{Y}/\hat{\mu }^2_\textrm{Y}/T$ in equation (11c) vanishes. For small *T* however, input uncertainty (in $Y_t$) does have an effect on LCOE averages — it increases the LCOE, especially for small *T* [cf. equations (4c) and (11c)], while small $Y_t$ uncertainty lets the LCOE curves for the O- and YO-scenario largely coincide. Also, the approximate character of equation (4c) becomes most apparent for large $Y_t$ uncertainty (i.e., in the wYO-scenario) and very small *T*. There, the standard approximation underestimates the LCOE. For larger *T*, we observe a quick convergence to ACME output as anticipated in the Methods section [cf. equation (12)].

As a consequence, the value of the calculated NPV does not hinge on the amount or shape of input uncertainty. In contrast, the projected LCOE is driven up by yield forecast uncertainty, particularly for short project lifetimes. For large yield forecast uncertainties and short project lifetimes, the standard approximation underestimates the LCOE and thus — by this measure — overestimates the economic feasibility of the PV project in question.

### Standard deviations

**Fig. 3:**
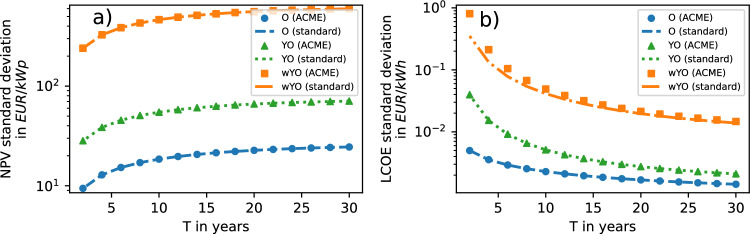
(**a**) NPV standard deviations and (**b**) LCOE standard deviations computed with ACME approach and standard approximation in three scenarios.

NPV uncertainty — as given by the NPV standard deviation — increases with *T* and with input uncertainty (Fig. 3a), as predicted by equation (11b). Values delivered by the ACME approach and the standard approximation moreover match exactly due to the exact character of equation (4b). In contrast, LCOE uncertainty decreases with *T* (Fig. 3b), in line with the prediction of equation (11d).

As in the NPV case, an increased input uncertainty is reflected by increasing LCOE uncertainty, with only the O-scenario yielding a perfect match of standard approximation and ACME predictions due to the linearity of equation (2) in $O_\textrm{R,t}$. As for LCOE averages, the standard approximation underestimates LCOE uncertainty in the wYO-scenario, but across a fairly large *T* interval, before convergence to ACME output.

Hence both NPV and LCOE forecasts follow intuition in that for them, input uncertainty is a driver of output uncertainty. Yet in contrast to the NPV case, the LCOE forecast accuracy actually *increases* with project lifetime and is moreover systematically overestimated by the standard approximation, especially for short project lifetimes.

### P90 values

**Fig. 4:**
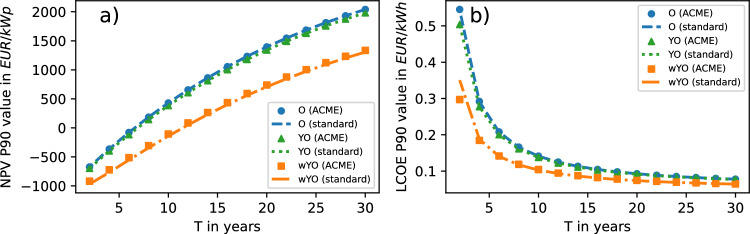
(**a**) NPV P90 values and (**b**) LCOE P90 values computed with ACME approach and standard approximation in three scenarios.

With NPV averages increasing faster with *T* than standard deviations (cf. equations (11a)-(11b) ), the monotonically increasing behaviour of NPV P90 values in Fig. 4 is plausible across all three scenarios of varying input uncertainty. Moreover, the higher the input uncertainty, the smaller the NPV P90 value for fixed *T*, which is a consequence of increasing NPV uncertainty (Fig. 3a) around constant NPV averages (Fig. 2a). We observe a fairly good agreement between ACME predictions and the standard approximation, with only large input uncertainty triggering a slight underestimation of P90 values by the standard approximation. The good performance of the standard approximation here can be attributed to averages and standard deviations coinciding with ACME predictions in Fig. 2a and Fig. 3a.

For similar reasons as in the NPV case, the monotonically decreasing *T*-dependency of LCOE P90 values in Fig. 4b follows from equations (11c)-(11d). Again we observe that, for fixed *T*, increasing the input uncertainty decreases the LCOE P90 value. The excellent fit of standard approximation P90 values and ACME output in Fig. 4b is counterintuitive, especially for small *T* in the wYO-scenario. This is because in this regime — and unlike in the NPV case — averages and standard deviations obtained from the standard approximation can differ significantly from ACME predictions (cf. Fig. 2b and Fig. 3b), with ACME PDFs being non-Gaussian (cf. Figs. 8c-i).

**Fig. 5:**
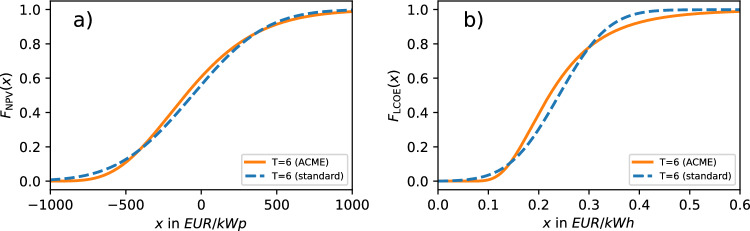
NPV cumulative distribution functions and LCOE cumulative distribution functions computed with ACME approach and standard approximation in the wYO-scenario.

This remarkable fit is put into perspective by plotting the cumulative distribution functions for small *T* in the wYO-scenario. In the resulting Fig. 5, we observe for both the NPV and LCOE case that percentiles of very small and very large rank are underestimated by the standard approximation, while being overestimated for intermediate percentile ranks. At the two intersections of ACME and standard approximation CDFs, computed percentiles coincide — for $T=6$ in the wYO-scenario, these are roughly the 20th and 85th percentile (Fig. 5a) or the 10th and 80th percentile (Fig. 5b), explaining the good fit for P90 values in Fig 4. The larger *T* and the smaller input uncertainty, the less ACME and standard approximation CDFs differ from each other, and the less pronounced are resulting percentile mismatches.

An added benefit of considering CDFs is that they directly deliver the probability of the target variable being in a given interval. For instance, in the wYO-scenario with $T=6$, the probability of the LCOE being between 0.1 EUR/kWh and 0.2 EUR/kWh is $F_\textrm{LCOE}(0.2\,\mathrm {EUR/kWh})-F_\textrm{LCOE}(0.1\,\mathrm {EUR/kWh})\approx 0.38$ in the ACME framework and 0.27 in the standard approximation (cf. Fig. 5b).

The consequences for investment decisions are multi-faceted: As in the case for NPV and LCOE averages, prolonging the project lifetime increases profitability when instead considering P90 values. Yet in the NPV case, the projected competitiveness decreases with input uncertainty while it increases in the LCOE case. Moreover, the competitiveness in the LCOE case is now *underestimated* by the standard approximation for large input uncertainty and short project lifetimes.

### Supplementary profitability metrics

**Fig. 6:**
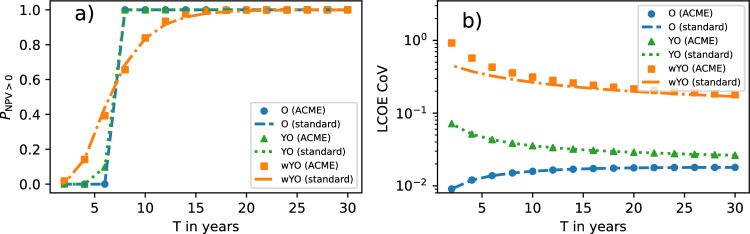
(**a**) Probabilities of profitability and (**b**) LCOE coefficients of variation computed with ACME approach and standard approximation in three scenarios.

Apart from the classical profitability metrics computed above, we can extend the economic analysis to two other quantities. It is straightforward to compute the probability of profitability $P_\mathrm {\textrm{NPV}> 0}\equiv 1-F_\textrm{NPV}(0)$. As expected, this probability is close to zero for small *T* and almost 1 for large *T* (Fig. 6a). The location of the transition between these two values (the NPV payback year) does not significantly depend on the degree of input certainty (cf. Fig. 2a). However, the transition is steeper for smaller input certainties, in line with Fig. 3a. It is only for large input uncertainties that the quality of the standard approximation’s predictions noticeably worsens.

Additionally, we calculate the LCOE’s coefficient of variation $\textrm{CV}_\textrm{LCOE}=\sigma _\textrm{LCOE}/\mu _\textrm{LCOE}$, which in our case is a measure of relative uncertainty in the computed LCOE. With both $\sigma _\textrm{LCOE}$ and $\mu _\textrm{LCOE}$ monotonically decreasing with *T* (cf. Fig. 2b and 3b), the resulting behaviour of the $\textrm{CV}_\textrm{LCOE}$ is not obvious. In Fig. 6b, we observe that $\textrm{CV}_\textrm{LCOE}$ is a monotonically increasing function of *T* if only input uncertainty in the numerator of the LCOE is involved (i.e., in the O-scenario). For sufficiently large input uncertainty in the LCOE denominator (i.e., in the YO- and wYO-scenario), we note instead a monotonic decrease of $\textrm{CV}_\textrm{LCOE}$ with *T*. Moreover, $\textrm{CV}_\textrm{LCOE}$ increases with input uncertainty, with the standard approximation significantly underestimating the ACME value for large input uncertainties and smaller *T*.

For PV investors envisioning a concrete business model for electricity monetization, Fig. 6a demonstrates that input uncertainty can significantly smear out the predicted NPV payback year both to earlier and later dates. Contrasting the results of Fig. 3b on absolute LCOE uncertainties, Fig. 3b suggests that the relative LCOE uncertainty actually increases with project lifetime if the yield forecast uncertainty is negligible compared to O&M cost uncertainty.

### Probability densities

**Fig. 7:**
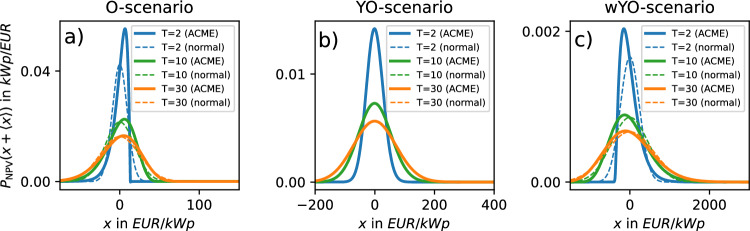
Centered ACME NPV probability density functions and normal distributions of same mean and variance. Computed for three scenarios and different lifetimes.

**Fig. 8:**
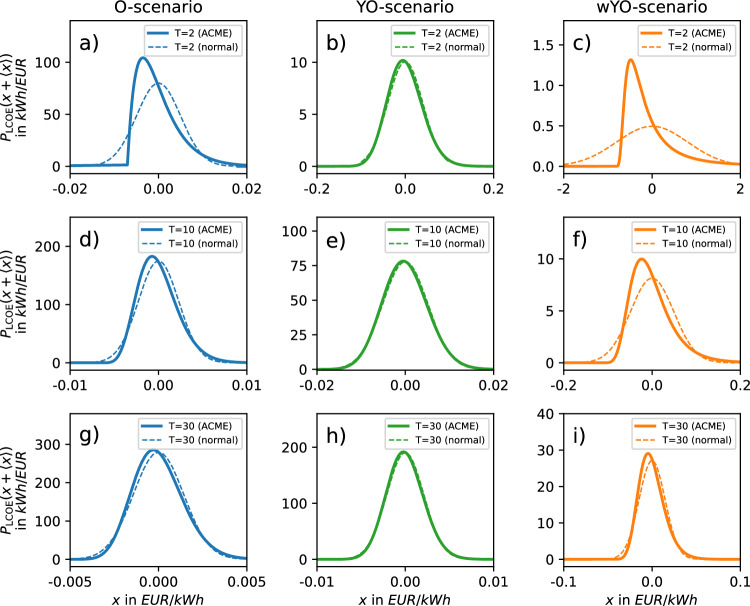
Centered ACME LCOE probability density functions and normal distributions of same mean and variance. Computed for three scenarios and different lifetimes.

The above discussion of computed NPV and LCOE quantities often invoked the purported shape of underlying PDFs. Here, we show the latter in Fig. 7 and Fig. 8 for the most relevant parameter regimes.

We observe that in the NPV case, PDF widths grow both with input uncertainty and *T*, whereas they decrease with *T* in the LCOE case (cf. Fig. 3a and Fig. 3b). The O-scenario (Fig. 7a and Figs. 8a,d and g) illustrates how heavily non-Gaussian input (drawn from a single exponential distribution) propagates in NPV and LCOE calculation. For small *T*, the resulting PDFs are heavily non-Gaussian, but become Gaussian for larger *T* when conditions for the central limit theorem are better met. To a weaker extent, these observations also apply to the wYO-scenario (Fig. 7c and Fig. 8c, f and i) that features a more heterogeneous non-Gaussian input drawn from an exponential distribution as well as a broad and time-dependent unimodal distribution. In the YO-scenario, Gaussian-shaped input propagates to Gaussian-shaped output, independently of the value of *T* (Fig. 7b and Fig. 8b, e and h).

The plots show that, in an investor-oriented model setup like ours, the Gaussian assumption for NPV and LCOE PDFs only holds in two cases: if the yield uncertainty is indeed given by a normal distribution or the projected lifetime surpasses 30 years. Consequently, caution should be exercised when leveraging the purported Gaussianity of LCOE and NPV variables for computational shortcuts in economic analysis (cf. Methods section).

## Conclusions

In this work, we perform a systematic study of uncertainty propagation in the NPV and LCOE analysis of PV plants. To this end, we introduce the ACME approach that traces — on the level of probability distributions — the uncertainty propagation from independent input variables to target variables. This is achieved through leveraging the occurrence of independent random variables in basic arithmetic operations occurring in NPV and LCOE, switching between different representations of probability distributions. The accuracy of the framework is limited only by its numerical implementation (for which we formulate several sanity checks), and its execution speed promises to be often much faster than that of Monte-Carlo simulations of similar accuracy. Computed ACME expressions tend to involve only low-dimensional integrals (relative to the number of considered input variables) that are more amenable to analytic treatment than the brute-force formulation of the problem.

We apply the ACME approach to different scenarios that reflect an investor’s perspective in the economic analysis of PV plants. These scenarios feature constant input averages, yet varying degrees of input uncertainty, and compute several quantifiers of the stochastic character of NPVs and LCOEs. This is done for a range of plausible PV plant lifetimes, with results qualitatively predicted by scaling relations and quantitatively compared with the output of a standard approximation framework. The analysis confirms the intuition that increased input uncertainty triggers increased output uncertainty (i.e., decreases forecast accuracy) in both the NPV and the LCOE case. Less intuitively, we observe that the effect of input uncertainty on economic analysis is not clear-cut: In the NPV case, the forecast economic competitiveness is independent of input uncertainty (according to NPV averages) or a decreasing function of it (according to NPV P90 values). In the LCOE case, the forecast competitiveness decreases or increases with (nonlinear) input uncertainty, depending on whether LCOE averages or P90 values are considered. Moreover, we observe that the forecast accuracy increases (NPV) or decreases (LCOE) with the assumed project lifetime. We notice that even for longer project lifetimes, non-Gaussian input can trigger non-Gaussian NPV and LCOE output, with the latter being rather the norm than the exception in our investor-centric analysis. In our study, the standard approximation and ACME approach give matching predictions whenever they should (i.e., for many years of operation as well as for any NPV average and standard deviation), and a fairly good agreement for all other quantities and regimes. The one exception are large uncertainties entering LCOE calculations nonlinearly, i.e., exactly the scenario where the assumptions of the standard approximation are violated. Observed discrepancies should hence also occur for other choices of input PDFs provided that the yield standard deviation is sufficiently large.

For a broader picture — beyond our input uncertainty scenarios and sensitivity analysis for *T* — of how input assumptions influence ACME and standard approximation output, we encourage further computational studies featuring comprehensive robustness checks. Future work could also elaborate on the inclusion of correlated input variables as briefly outlined in the text, and determine whether the relatively good fit between the two discussed frameworks persists in that case. Moreover, ACME and standard approximation could be applied to other contexts like the PV performance modeling chain, with the added difficulty of tracing uncertainty propagation through a sequence of submodels, some of which are only given in implicit form.

## Data Availability

All data generated during this study is included in this published article.
